# External cervical resorption—a review of pathogenesis and potential predisposing factors

**DOI:** 10.1038/s41368-021-00121-9

**Published:** 2021-06-10

**Authors:** Yiming Chen, Ying Huang, Xuliang Deng

**Affiliations:** Department of Geriatric Dentistry, Peking University School and Hospital of Stomatology & National Center of Stomatology & National Clinical Research Center for Oral Diseases & National Engineering Laboratory for Digital and Material Technology of Stomatology & Beijing Key Laboratory of Digital Stomatology & Research Center of Engineering and Technology for Computerized Dentistry Ministry of Health & NMPA Key Laboratory for Dental Materials, Beijing, China

**Keywords:** Oral diseases, Risk factors

## Abstract

External cervical resorption (ECR) refers to a pathological state in which resorption tissues penetrate into the dentin at the cervical aspect of the root. Despite being latent in its initial phase, ECR could cause severe damage to mineralized dental tissue and even involve the pulp if not given timely diagnosis and treatment. Nevertheless, the etiology of ECR is still poorly understood, which adds to the difficulty in early diagnosis. ECR has received growing attention in recent years due to the increasing number of clinical cases. Several potential predisposing factors have been recognized in cross-sectional studies as well as case reports. In the meantime, studies on histopathology and pathogenesis have shed light on possible mechanisms of ECR. This review aims to summarize the latest findings in the pathogenesis and potential predisposing factors of ECR, so as to provide pragmatic reference for clinical practice.

## Introduction

Root resorption is featured by progressive degradation of mineralized dental tissue due to overactivity of absorptive cells.^1^ Depending on whether the outcome is desirable or not, root resorption can be classified into physiologic and pathologic resorption, the former of which generally refers to root resorption during exfoliation of deciduous teeth.^2^ Based on the location of the lesion, pathologic resorption is segmented into external root resorption (ERR) and internal root resorption (IRR).^3^ Classifications and terminology regarding external resorption vary across different studies. According to the Andreasen Classification, which is by far the most frequently cited in literature, external resorption can be further categorized as inflammatory, replacement, and surface resorption.^2^

External cervical resorption (ECR) was previously regarded as a particular type of progressive inflammatory resorption related to bacterial infection.^4,5^ In recent years, histopathological findings have indicated that ECR possesses several distinct features compared with inflammatory resorption.^6,7^ To be specific, ECR initiates at the cervical aspect of the tooth, which is beneath the epithelial attachment.^6,8^ By comparison, inflammatory resorption could occur at any section of the root.^3^ Besides, the distribution of the resorption lesion is also a pathognomonic feature of ECR.^9^ Concretely speaking, as ECR proceeds, resorptive tissues extend circumferentially or horizontally in the dentin while leaving the pulp intact.^10^

ECR was used to be considered as a rare disease due to a lack of case reports. Its prevalence rate varied from 0.02% to 0.08% according to different epidemiological studies.^10,11^ Multiple cervical root resorption (MCRR) is a peculiar form of ECR in which three or more teeth are implicated.^12^ Despite its destructive nature, early stages of ECR lack apparent symptoms, which poses a challenge for clinicians.^1,13^ In most cases, it is not until when the resorption reaches its advanced phase that symptoms begin to develop.^6^ Consequently, ECR may well have caused irreversible damage to the tooth structure upon its being diagnosed.^1,13^

In consideration of its undesirable outcome, clinicians have been attaching greater significance to early diagnosis and prevention of ECR in order to contain the disease ahead of extensive progression. Nevertheless, until recently the majority of literature regarding ECR were case reports rather than studies on its pathologenesis.^14^ As a result, our knowledge concerning the etiology of ECR primarily derives from sporadic reports of clinical cases, which have put forward various predisposing factors. This stresses the necessity of summarizing its uncovered etiology so as to provide reference for further research. Thus, this article will review the latest findings in the pathogenesis as well as potential predisposing factors of ECR.

## Pathogenesis

The low morbidity of ECR has led to a lack of studies on its pathogenesis, which makes ECR by far one of the least understood types of root resorption.^1,14^ Our current understanding of its etiology is mainly based on histopathological findings. In the meantime, significant improvements are still to be made concerning the precise mechanism of ECR. As thus, the latest position statement by European Society of Endodontology described the etiology of ECR as poorly understood.^14^

In general, studies on the pathogenesis of ECR primarily focus on two basic aspects, namely the lesion’s histopathological manifestation as well as the cellular and molecular mechanisms of osteoclastogenesis.

### Histopathology

**Fig. 1:**
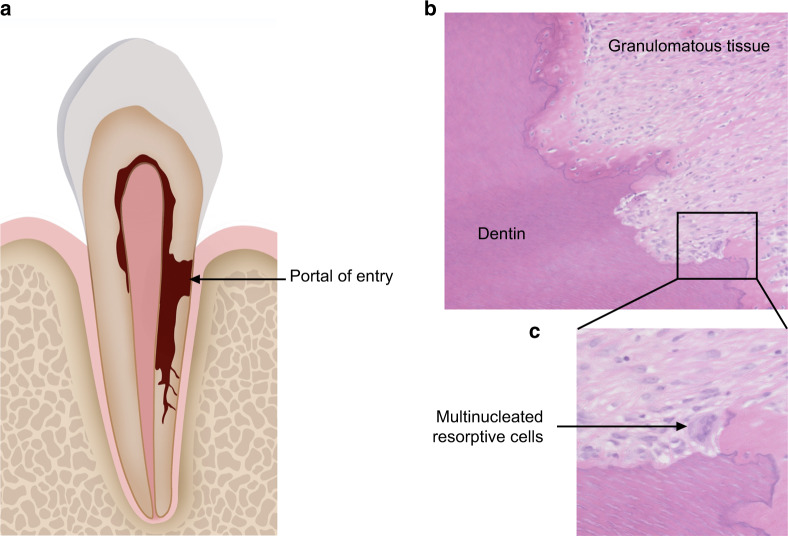
Schematic image and histological sections of ECR. **a** Schematic image presenting the histopathological patterns of ECR lesion. **b** Pathological section of ECR lesion showing granulomatous tissue and the impaired dentin. **c** Pathological section of ECR lesion showing multinucleated resorptive cells located in resorption lacunae

Pathological sections have provided important reference for understanding the cellular as well as tissue constituent of the resorption lesion.^7,13,15^ ECR is believed to derive from a disruption of the periodontal ligament, which subsequently induces inflammation.^16^ Following the infiltration of inflammatory cells, granulation tissues form and penetrate into the dentin.^7^ The resorption lesion extends circumferentially and apico-coronally, thereby forming multiple resorption channels inside the root (Fig. 1a).^7^ The resorption tissue is primarily consisted of blood vessels, fibrous tissue, and a variety of cellular components including fibroblasts, vascular endothelial cells, adipocytes, and leukocytes (Fig. 1b). Multinucleated cells which are morphologically similar to osteoclasts form within the resorption lacunae (Fig. 1c).^7^ As resorption proceeds, the lesion is partially repaired by ingrowth of osseouslike tissue.^7^

Besides providing insight into the resorption process, histopathological studies have also shed light on vital structures related to the pathogenesis of ECR. Upon initiation of resorption, granulation tissues penetrate into the dentin at the cervical aspect of the root (Fig. 1a).^1,13^ In most cases, regardless of the extent of the lesion, the portal of entry is confined to the level of the cemento-enamel junction (CEJ).^8^ This unique feature of ECR has raised the concern over heterogeneity in the susceptibility to resorption of different types of mineralized dental tissue.^17,18^ During the degradation of osseous tissue, the outer layer of nonmineralized osteoid must be dissolved by protease before osteoclasts can attach to the mineralized matrix and initiate resorption.^19^ With regards to dental hard tissue, the surface of the root is covered by a layer of cementoid, which plays a similar role as the osteoid layer in resisting resorption.^2^ Epidemiological studies point out that hypo-mineralization of the cementoid increases the risk of ECR.^10^ Besides, histological sections reveal that cementoid is mainly distributed in the apical and middle section of the root.^20^ Therefore, the lack of cementoid at the cervical aspect could be a reason for its susceptibility to resorption. Apart from acting as a barrier against resorption tissues, cementum could also react to external stimulus by enhancing the repairment process. To be specific, cementocytes react to resorption stimulation by reducing the expression of sclerostin, a protein which could inhibit osteogenesis.^21^ Thus, the cementum is considered to be pivotal in protecting mineralized dental tissue against ECR. Developmental defects at the CEJ which involve the cementum is regarded as a major predisposing factor of ECR.^7^ Optical microscopy as well as scanning electron microscopy both point to the discovery of a gap between the dentin and the cementum at the CEJ of normal teeth.^22^ The occurrence rate of this developmental defect in the population has been reported as ~10% in studies.^23^ Under higher amplification, small holes representing dentin tubules were detected in the gap. Researchers therefore put forward the concept of “*dentin-cemento-enamel junction*” so as to vividly depict the correlation of dental hard tissues at the cervical section.^22^

### Cellular and molecular mechanisms of ECR

Different terms have been put forward with regards to the resorptive cells that actively degrade mineralized dental tissue in ECR. As cells taking part in the shedding of deciduous teeth are referred to as *odontoclasts*, several studies of ECR have adopted this name.^1,13,24,25^ Meanwhile, some other studies prefer using the term *osteoclasts*.^3,9^ In view of the diverse perspectives on terminology, some experts have suggested using the term *osteoclast like cells* or *clastic cells*, so as to draw a distinction between these peculiar resorptive cells and osteoclasts.^7,14^

The underlying mechanisms of osteoclastogenesis in ECR is a key issue concerning its cellular mechanisms. Compared with other types of mineralized dental tissue, the dentin possesses a higher content of non-collagenous bone matrix proteins, which creates a favorable environment for resorptive cells to adhere.^17^ Osteocalcin and Osteopontin, which are expressed at high levels in the dentin, facilitate osteoclastogenesis by acting as chemo-attractants.^18^ On top of that, bone sialoprotein (BSP) possesses an arginine-glycine-aspartic acid (RGD sequence), which can be recognized by αvβ3 vitroneilin receptors of resorptive cells.^26^ The binding process induces the adherence of osteoclasts to the root surface, which is an essential step in resorption initiation.

Histopathological studies suggest that osteoclastogenesis occurs posterior to leukocyte infiltration.^7^ Yet, the exact cause of inflammatory response in ECR remains controversial.^27^ By far, scholars have proposed two hypotheses with regard to the activation of osteoclastogenesis, namely the inflammation theory, and the infection theory.

#### The inflammation theory

This theory proposes that resorption is initiated due to non-infectious exogeneous stimulation, which induces local inflammation in the periodontium.^27–29^ Histopathological studies have revealed that microorganisms are mainly distributed in the peripheral region of the resorption cavity, which indicate that microbe infection occurs posterior to the activation of osteoclastogenesis.^7,30^ Therefore, infection may not be a requisite in the initiation of ECR.

#### The infection theory

This theory considers the mechanism of ECR to be similar to that of periodontitis, which is to say the presence of microorganisms is essential in the initiation of osteoclastogenesis.^4,31^ Since microbe infection has been proved to aggravate leukocyte infiltration and induce bone resorption in periodontitis, it is reasonable to infer that microorganisms may play a similar role in the initiation of osteoclastogenesis in ECR.^32^

Despite there being diverse perspectives on the initiation of ECR, inflammatory response is indispensable for osteoclastogenesis.^32^ Extensive research has been carried out on the correlation between root resorption and inflammatory response. Interestingly, proinflammatory cytokines holding the key to ECR are similar to those generated during external inflammatory resorption (EIR) and periodontitis.^33,34^ Therefore, ECR may share a common mechanism with other types of mineralized tissue resorption.^35^

Under physiological conditions, bone resorption is strictly regulated by systemic hormones and local factors so as to form a balance with bone formation.^36,37^ ECR occurs when the balance is tipped by local stimulation.^38^ In this case, hematopoietic stem cells (HSCs) undergo a succession of differentiation and form circulating peripheral blood monocytes as well as tissue macrophages, which ultimately fuse into mature multinucleated resorptive cells.^37^

Studies have revealed that receptor activator of nuclear factor κB ligand (RANKL) and osteoprotegerin (OPG) are two key factors in modulating bone metabolism. The former acts as an agonist of osteoclastogenesis, while the latter is regarded as an inhibitor.^39^ In other words, the OPG/RANKL ratio is negatively correlated with the activation of osteoclastogenesis.^32^ The differential expression of OPG and RANKL is considered as an underlying reason for the heterogeneity of susceptibility to resorption between cementum and dentin. Compared with the dentin, the OPG/RANKL ratio is higher in the cementum, which results in its stronger resistance against resorption.^21^

Several cytokines are related to the regulation of RANKL expression, among which IL-1β, IL-6, and TNF-α are recognized as key factors contributing to its upregulation by studies on both root resorption and periodontitis.^32,40–42^ Given that the initiation of osteoclastogenesis in root resorption and in periodontitis share a similar mechanism, it is reasonable to infer that resorptive tissues might derive from periodontal tissues in ECR.^33,34^

Macrophages are also believed to play an important role in regulating osteoclastogenesis.^43^ External stimulus could alter the ratio of different phenotypes of macrophages. To be specific, an increase in the M1 phenotype, which is commonly accompanied by upregulation of IFN-γ and TNF-α, is positively correlated with osteoclastogenesis in both periodontitis and external resorption. Removal of the external stimulus could result in an increase in the M2 phenotype and a decrease in the M1 phenotype, which in turn inhibits root resorption.^44,45^

In addition to local inflammatory response, systemic factors also play an essential role in regulating bone metabolism. Hormones regulating blood calcium concentrations pose a significant impact on osteoclastogenesis.^46^ Parathormone and 1,25-dihydroxyvitamin D3 have been shown to promote osteoclast formation and activity, while calcitonin, estrogen, thyroxine, and prostaglandin E2 impose an opposite effect.^47–49^ The increased morbidity of osteoporosis among post-menopausal women is believed to be related to hyper-activation of osteoclasts.^50^ Several case reports have presented results of screening tests of serum endocrine levels. However, significant fluctuations of hormone levels are rarely seen among ECR patients, and only a small number of case reports have described abnormal laboratory examination results.^51–53^ Thus, the relevance between root resorption and hormone levels calls for further investigation.

Research on the specific mechanisms of ECR primarily focus on the activation of certain cellular pathways as well as abnormalities of gene expression. A study on the differential expression of micro-RNAs shed light on the activation of the mineral resorption pathway as well as the neurotrophic signaling pathway during ECR.^54^ Compared with nonaffected gingival connective tissues adjacent to the resorption lesion, the expression of miR-122-5p, whose target gene functions in vesicular transport of proteins as well as cell apoptosis was significantly up-regulated in the diseased granulomatous tissue. On top of that, expressions of miR-20a-5p, miR-30b-5p, miR-210-3p, and miR-99a-5p were reduced. Concretely speaking, miR-20a is regarded as a key factor in osteogenic differentiation of human mesenchymal stem cells.^55,56^ Besides, studies show that miR-99a expression is negatively correlated with inflammation and in the meantime results in a reduction of the M1/M2 ratio.^57^ In view of the indispensable role of macrophages during osteoclastogenesis, miR-99a-5p could be a vital regulating factor of ECR. On top of that, the expression of miR-30b-5p was found to be down regulated in both ECR and chronic periodontitis (CP). Studies have shown that miR-30b-5p is capable of suppressing the expression of inflammatory mediators such as IL-1β.^58^

In another recent study, whole exome sequencing on a two-generation family with four members diagnosed with ECR was carried out. The results revealed a mutation in the interferon regulatory factor 8 gene (IRF8^G388S^).^59^ Notably, in vitro studies have indicated that the IRF8^G388S^ variant is capable of facilitating osteoclastogenesis. Moreover, IRF8 deficiency mice has been shown to exhibit an osteoporosis phenotype, along with an increase in osteoclast quantity and resorption activity.^60^ Notably, the number of osteoclasts lining the alveolar bone in IRF8 deficiency mice increased without external inflammatory challenge.^60^

## Potential predisposing factors

By far, the majority of studies on the etiology of ECR has been focusing on potential predisposing factors. Apart from retrospective studies, case reports on ECR have also put forward several possible risk factors.^10,52,61^ Nevertheless, despite the increasing number of risk factors identified in studies, the underlying correlation between these factors and occurrence of ECR remains undefined.^61^ In view of the uncertainty of its pathogenesis, summarizing the potential predisposing factors may provide significant reference for the prevention and early diagnosis of ECR.

### Overview of previous cross-sectional studies

**Fig. 2:**
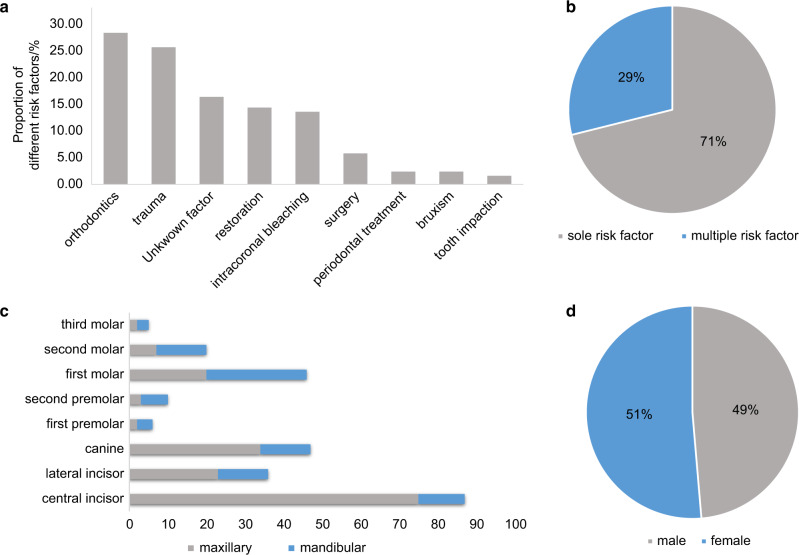
Potential predisposing factors and distribution of ECR presented in a cross-sectional study carried out in Australia.^10^ **a** Proportion of different risk factors identified in patients. **b** Comparison of the percentage of patients detected with sole risk factors and multiple risk factors. **c** Distribution of teeth diagnosed with ECR. **d** Distribution of patients’ gender

**Fig. 3:**
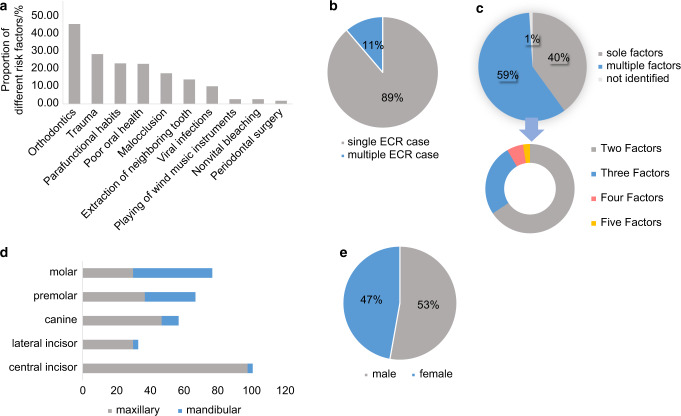
Potential predisposing factors and distribution of ECR presented in a cross-sectional study carried out in Europe.^61^ **a** Proportion of different risk factors identified in patients. **b** Comparison of the percentage of patients diagnosed as ECR and multiple ECR. **c** Comparison of the percentage of patients detected with sole risk factors and multiple risk factors. **d** Distribution of teeth diagnosed with ECR. **e** Distribution of patients’ gender

**Fig. 4:**
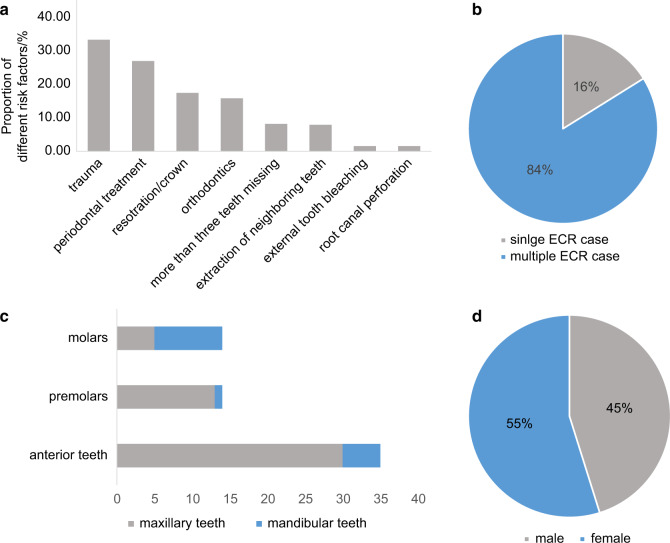
Potential predisposing factors and distribution of ECR presented in a cross-sectional study carried out in Asia.^52^ **a** Proportion of different risk factors identified in patients. **b** Comparison of the percentage of patients diagnosed as ECR and multiple ECR. **c** Distribution of teeth diagnosed with ECR. **d** Distribution of patients’ gender

In spite of the diverse perspectives on its pathogenesis, studies on the distribution and potential predisposing factors of ECR have reached similar conclusions. By far, three cross-sectional studies focusing on the distribution and potential predisposing factors have been carried out in Australia (1999), Europe (2017), and Asia (2020), respectively.^10,52,61^ No significant gender difference has been reported among ECR patients (Figs. 2d, 3e, 4d).^31,62^ However, with regard to MCRR, different studies point to diverse conclusions, which could be the result of bias due to small sample sizes.^63^ A systematic review on multiple idiopathic cervical resorption pointed out that younger females, especially Caucasians, are more susceptible to MCRR, while another research claimed that the incidence rate was higher in men.^52,63^ So far, the majority of ECR cases are sporadic, with merely one report pointing to familial inheritance.^64^

ECR showed a wide age distribution across all studies, while the majority of patients were young and middle-aged ranging from 20 to 50 years old.^10,52,61^ Concretely speaking, the susceptible age is related to specific risk factors. In cases of para-functional habits, the highest frequency of appearance was identified in patients aged 35–39. While concerning poor oral health, it was mostly observed in patients above ages 65. Orthodontics showed a high incidence in ages between 15 and 19.^10,52,61^

With regards to the distribution of affected teeth, ECR showed a high incidence rate in maxillary teeth, among which maxillary incisors and canines presented the highest susceptibility (Figs. 2c, 3d, 4c).^10,52,61^ This could be attributed to a higher incidence rate of dental trauma in the maxillary dental arch.^65^ Besides, anterior teeth are subjected to greater movement during orthodontic treatment due to their being located at the apex of the dental arch, which increases the risk of post-orthodontic resorption.^10^ Apart from maxillary teeth, mandibular molars also showed high susceptibility to ECR in several retrospective studies, which could possibly be caused by extraction of neighboring teeth.^61^ Therefore, it is reasonable to conclude that susceptibility to ECR is correlated to tooth position.

In terms of potential predisposing factors, trauma and orthodontic treatment were recognized as major risk factors among all three cross-sectional studies, while periodontal surgery, restoration, and bruxism were also identified as common risk factors (Figs. 2a, 3a, 4a).^10,52,61^ Nevertheless, due to regional differences and the time span between studies, there appeared to be a few significant variations in the proportion of each factor.

**Fig. 5:**
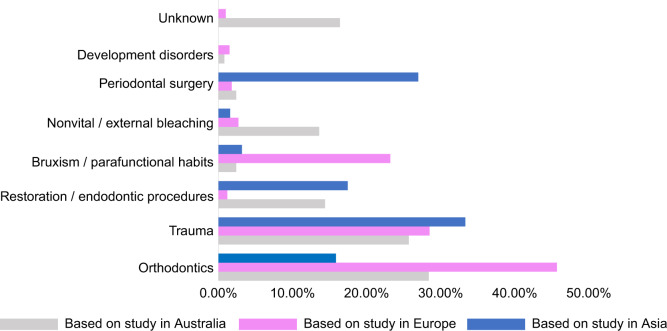
Overview of major predisposing factors identified in three cross-sectional studies^10,52,61^

A noteworthy difference appeared in the detectable rate of predisposing factors. According to the first cross-sectional study which was carried out in Australia, 16.4% of all examined teeth had no confirmable potential predisposing factor.^10^ In the two recent studies, risk factors were identified in almost all examined teeth (Fig. 5).^52,61^ Moreover, due to modifications in treatment methods, the incidence rate of post-bleaching ECR significantly decreased over the past two decades.^10,61^ The proportion of different factors varied across regions as well. To be specific, Asians showed a 10 times higher incidence rate of prior periodontal treatment along with an apparently smaller percentage of malocclusion, compared with Europeans (Fig. 5).^52,61^ Furthermore, a latest cross-sectional study in Asia showed that 83.87% of patients were diagnosed as MCRR, while the percentage was merely 11.3% in European patients (Figs. 3b, 4b).^52,61^ This has drawn particular attention as MCRR was previously considered as a relatively uncommon type of ECR.

Besides divergence in the constituent ratio of risk factors, new studies also proposed that a considerable percentage of cases were related to multiple risk factors (Figs. 2b, 3c). To be specific, 59% of cases were identified as multifactorial in the study carried out in Europe (2017), while the proportion was 28.9% in the Australian study (1999).^10,61^

It was not until recently that the first retrospective case control study on local and systemic predisposing factors of ECR was carried out.^66^ Compared with previous epidemiological studies which were cross-sectional,^10,52,61^ case control studies offer a superior level of evidence.^67^ A noteworthy finding was that among the six major predisposing factors (bruxism, trauma, eruption disorders, extraction of an adjacent tooth, orthodontic treatment, and restoration) taken into account, trauma was the only factor which showed a significant difference between the ECR group and the control group.^66^ However, in view of the study’s small sample size as well as its higher age distribution, the researchers did not draw valid conclusions regarding the differences between the relevance rate of post-orthodontic ECR.^66^

### Major predisposing factors

#### Orthodontics

Orthodontics is a commonly identified risk factor not only in ECR, but also in other types of external resorption.^68,69^ In terms of its incidence rate, recent studies revealed a sharp increase from 24.1% to 45.7% during the past two decades, which is primarily ascribed to the rise in the number of patients seeking orthodontic treatment.^61^ Nevertheless, a latest epidemiological study on Asians claimed that orthodontics was only the forth most common predisposing factor, accounting for merely 15.87% of the population.^52^ This might be attributed to the divergence in craniofacial patterns and variations in perception of esthetics.^70,71^

Maxillary anterior teeth, especially maxillary canines and maxillary central incisors, showed a high susceptibility to ECR during and after orthodontic treatment. It is generally held that canines are more resistant to orthodontic movement compared with other teeth. Besides, as incisors are located in the apex of the dental arch, they are subjected to greater tooth movement during the treatment process. Both factors could possibly lead to excessive force exertion, which causes greater damage to the root. In addition, as mandibular molars are often used as anchorage teeth, they also showed a high incidence of resorption. Case reports have shown that the appliance of class II elastics led to higher susceptibility to root resorption.^72,73^ Given that these elastics are attached to maxillary canines and mandibular first molars, the root surface may be exposed to greater forces, which thereby increases susceptibility to resorption.^10^

Systematic reviews pointed to a positive correlation between the extent of root resorption and the amount of force exerted on the teeth.^74^ Besides, extending the time of acting force also increased the severity of root resorption. As a preventive measure, intermittence during tooth movement, which enables regeneration of osseous tissue, is considered effective in mitigating the resorptive process.^75^ According to previous studies, most scholars reached consensus on the fact that the direction of tooth movement and the loading regimen (continuous vs. intermittent forces) both have considerable impact on the resorption process.^74^ Observations show that applying 1 N of force to the cervical section of the root for over 2 months could provoke severe resorption.^76^ Therefore, maintaining an “optimal” orthodontic force within the levels of capillary pressure during orthodontic treatment is necessary for reducing the risk of ECR.^76^

The role of orthodontic forces in inflammatory root resorption has been well-explored. It is possible that post orthodontic ECR might share a similar mechanism. As a stimulation to the immune microenvironment, orthodontic forces induce the synthesis of IL-1β and IL-6, both of which are pivotal proinflammatory cytokines in initiating osteoclast activation.^34^ Furthermore, applying excessive orthodontic force could lead to the collapse of capillaries in the PDL. This results in the dysfunction of blood supply, and thereby creates a hypoxia microenvironment.^77^ As a consequence, the activity of cementoblasts is repressed, while in the meantime osteoclasts are activated. Once the protective layer of cementum is degraded, inflammatory response is amplified due to exposure of RGD peptides.^78^

It is worth noting that despite sharing a similar pathogenesis with inflammatory resorption, ECR does not occur immediately after orthodontic treatment. An interval of several years is commonly seen in the majority of cases, during which other predisposing factors may also contribute to the initiation of ECR.^1,72^ This may explain the reason why orthodontics is seldomly presented as a sole risk factor among ECR patients.^61^ Besides, further analysis discovered a synergy of orthodontics and other risk factors in inducing ECR, such as trauma, extraction of a neighboring tooth, and parafunctional habits.^61^

#### Trauma

Trauma has long been considered as a prominent risk factor of root resorption. Epidemiological research on European populations revealed that nearly one-third of patients with ECR recalled a history of trauma, which is second only to orthodontics.^61^ Besides, a recent study carried out in Asia showed trauma as the leading cause of ECR.^52^ In terms of acute injury, the majority of ECR cases were detected within 2–5 months following trauma.^79^ Nonetheless, in cases of chronic dental trauma, the time lag between injury and resorption initiation is usually long. In addition, the medical history of dental trauma is largely based on the patient’s recalling. As a consequence, the incidence rate of post traumatic ECR might be underestimated.^1^

Similar as the mechanism of post-orthodontic ECR, damage to the surface of the root by traumatic injuries causes loss of cementum, which exposes the dentin to osteoclasts.^7^ The cervical region of the root is especially vulnerable to trauma due to its lack of cementoid.^20^ On the one hand, exposure of dentin at the CEJ triggers the activation of osteoclasts.^18^ On the other hand, absence of cementum allows intracanal bacteria and their endotoxins to reach the PDL more readily, which amplifies the inflammatory process.^80^ On top of that, trauma could alter the microstructure of the root surface, thereby creating an ideal surface for the adherence of osteoclasts.^52^

Dental injury occurs most frequently in youngster, which is in accordance with the predilection age of post-traumatic ECR.^81^ Its incidence rate also increases in proportion to the interval between occurrence of trauma and initial examination.^82^ Post-traumatic ECR is more likely to be detected in anterior teeth, among which maxillary central incisors are most frequently affected.^10^ This is consistent with the assumption that the location of the tooth in the dental arch has a direct influence on its susceptibility to trauma.^83^ To be specific, anterior teeth are more prone to dental trauma, while posterior teeth are more prone to orofacial trauma.^52^

Retrospective studies reveal that luxation poses a greater threat to root resorption compared with intrusion.^82^ In order to minimize damage to the CEJ, careful re-positioning of the luxated tooth along with re-adaptation of associated bone and soft tissues is essential.^84^ Furthermore, orthodontically repositioning the luxated tooth resulted in better marginal bone healing compared with surgical replacement.^85^ As stated in some cases, immature permanent teeth may possibly retain there pulpal vitality through appropriate surgical re-positioning after intrusive luxation.^84^ During the stage of mixed dentition, intrusion of primary teeth could cause developmental defects to the permanent successor, which may become a predisposing factor of ECR. This phenomenon is of particular concern when direct impairment of the unerupted successor’s cervical region is induced by the root apices of primary teeth.

#### Periodontal treatment

Consisting of sub-gingival scaling and root planing, as well as mucogingival surgery, the effect of periodontal treatment on ECR was often neglected in previous studies.^1^ However, a recent research revealed that up to 27% of the patients enrolled in the study claimed to have previously underwent periodontal treatment.^52^

Several studies have pointed out that scaling and root planing could result in alteration to the root surface.^86,87^ Despite the reduction of collateral impairment due to improvement in instruments, partial damage to the cementum is inevitable.^88^ Consequently, the exposure of dentin leads to activation of osteoclasts, which initiates root resorption. Moreover, microbial stimulation along with inflammatory response caused by periodontitis may also contribute to the activation of osteoclasts.^52^

During recovery, subsequent growth of epithelial cells along the root surface facilitates the forming of a protective barrier against osteoclasts.^89^ However, there is chance by which other types of periodontal tissue adheres to the root surface in advance of epithelium. This could possibly occur during guided tissue regeneration (GTR), a therapy of which the aim is to form a new layer of parodontium covering the root surface. Under these circumstances, mononuclear cells originating in the PDL may differentiate into osteoclasts and eventually lead to root resorption.^90^ ECR has been reported to occur posterior to GTR in several cases, some of which revealed a combination with ankylosis.^90–92^

#### Intra-coronal bleaching

As the first predisposing factor discovered by clinicians,^28^ intra-coronal bleaching was identified as the third largest risk factor in previous epidemiological researches. Maxillary incisors were considered to be most vulnerable to bleaching.^10^ However, in recent years, studies have shown a significant decrease in the incidence rate of ECR caused by bleaching. This could primarily be attributed to the standardization of bleaching protocols as well as improvements in bleaching agents.^61^ The latest cross-sectional study on predisposing factors found no cases related to internal bleaching.^52^

It is generally held that physical and chemical properties of the bleaching agents, as well as permeability of the dentin are the three major factors contributing to the occurrence of post-bleaching ECR. To be specific, the thickness of cervical dentin, the diameter of dentin tubules, the presence or absence of smear layer, and the temperature of bleaching agents codetermine the extent of permeability.^22^ Furthermore, a gap between the cementum and dentin at the cervical region enables bleaching agents to reach the PDL more readily. Several in vivo experiments have proved the bleaching agents’ being capable of penetrating through dentinal tubules.^93–95^

As the most commonly used bleaching agent, hydrogen peroxide (H_2_O_2_) can be applied either as an aqueous solution in different dilutions or as a paste combined with sodium perborate. Due to its capability of degrading mineralized and organic components, H_2_O_2_ is believed to reduce dentin hardness drastically.^96^ Besides, H_2_O_2_ is considered as an unstable solution, especially when brought into contact with catalase existing in oral environment. Thus, various kinds of free radicals could be released during the bleaching procedure, some of which could possibly dissolve mineralized dental tissue. Some researchers claim that resorption is sustained by the toxicity of byproducts of hydrogen peroxide.^97,98^ Apart from causing direct damage to the root, bleaching agents are also capable of activating osteoclast differentiation once they diffuse through the dentinal tubules and come into contact with the PDL.^28^ In addition, the forming of an acidic environment by bleaching agents provide a favorable environment for the activating of osteoclasts.^99^

Dental trauma combined with intracoronal bleaching led to a noticeable increase in the morbidity of ECR, which points to a correlation between the two factors.^10^ For one thing, dental trauma may well result in damage to the cervical cementum. For another, reparative cementum that forms after trauma attaches loosely to the dentin. Hence, trauma facilitates the diffusion of bleaching agents, and thereby amplifies the inflammatory response.

Referring to the prognosis, post-bleaching ECR may ultimately lead to ankylosis. Given that the periodontal tissue is likely to be devitalized after bleaching, the absence of PDL cells gives rise to the ingrowth of alveolar bone cells, which in turn facilitates ankylosis.^100^

Given the potential risk of bleaching, careful examination should be carried out before embarking on internal bleaching treatment. Probing and radiologic examinations are necessary for detecting defects in the cervical region. Coronal sealing of the root canal with glass ionomer cement serves as a feasible way of reducing the possibility of periodontal or cervical leakage.^84,101^

In recent years, the introduction of sodium perborate mixed with water as a substitute for hydrogen peroxide has brought about a considerable decrease in the morbidity of post-bleaching ECR.^102^ 35% carbamide peroxide showed a combination of sodium perborate’s safety and hydrogen peroxide’s efficacy in clinical practice.^101^

#### Surgery

Referring primarily to extraction of neighboring teeth, the incidence rate of post-surgery ECR ranged from 6.3% to 14% in different epidemiological studies.^10,61^ Collateral damage to the CEJ during surgery serves as a possible explanation to its mechanism.^1^ Due to the fact that extraction is more commonly seen in molars, the incidence rate of post-surgery ECR is higher in posterior teeth. In view of the overlapping of factors, more nuanced classifications of predisposing factors have been put forward by scholars in recent studies. To be specific, surgery has been subdivided into teeth extraction, teeth transplantation, surgical exposure of unerupted teeth, orthognathic surgery, and periodontal surgery.^1,61^ As a result, it would be difficult to compare the incidence rate of risk factors concerning surgery across different cross-sectional studies since the classifications adopted by these researches are not completely consistent.

#### Malocclusion

Malocclusion refers to either premature contact or overloading of the occlusal, both of which could lead to constant excessive force on the root. Consequently, excessive force may cause damage to the cementum, and thereby increases the root’s susceptibility to external resorption. Malocclusion was identified in 17.5% of all examined teeth in a recent research.^61^ However, the difficulty in clinical detection of malocclusion has caused several studies to overlook this risk factor.

#### Parafunctional habits

A recent study showed that parafunctional habits were identified in 23% of all the examined teeth, most of which were found in combination with other factors. Besides, patients aged 35–39 were most likely to be found with parafunctional habits.^61^ Similar as malocclusion, parafunctional habits are usually discovered through inquiry or by oral examination, and could easily be neglected. Therefore, the incidence rate of these two factors might be underestimated.

#### Undesirable oral hygiene

Poor oral health was considered as a predisposing factor with an incidence rate of 22.9% in a recent epidemiological study.^61^ However, it is notable that undesirable oral hygiene is seldomly identified as a sole risk factor, and was not presented in other retrospective studies. This is in accordance with histological findings which indicate that bacteria are predominately located at the outer layer of the resorption cavity. Accumulation of bacteria due to periodontitis or plaque could act as a driving force in sustaining the resorption process through increasing the expression of inflammatory cytokines such as IL-1β and MMP-1.^103^ In other words, poor oral health may possibly aggravate the resorption process under the premise of other predisposing factors. Thus, it is still uncertain whether bacterial infection alone could cause ECR.

#### Restorations

Restorations were presented as a major risk factor in several cross-sectional studies. However, due to a lack of case reports, the correlation between restoration and ECR is poorly understood. Previous studies revealed that intracoronal restorations accounted for the largest proportion of patients, while recent research indicated that crown protheses were also identified as a risk factor.^10,52^ Further study is required to clarify and define the respective fatalness of different types of restorations.

#### Viral infection

The correlation between viral infection and ECR has been depicted in a series of case reports, among which feline herpes virus type 1 (FeHV-1) takes up the largest proportion. Feline odontoclastic resorptive lesions (FORL), which is featured by resorption of the cervical dental tissue, is a common disease among domestic, captive, and wild cats. Notably, the clinical, radiologic, and histopathologic characteristics of ECR and FORL appear to be analogous.^104^ Besides, there has been a number of cases reporting patients with ECR having direct contact with domestic cats, some of whom showed positive titers of neutralizing antibodies against FeHV-1. Moreover, contact with cats appeared as the only identified predisposing factor in some cases. However, the exact effect of FeHV-1 on root resorption remains unclear. Some researchers point out that the virus might indirectly stimulate osteoclastogenesis via immunologic cells.^105^ Besides FeHV-1, varicella zoster virus (VZV) and hepatitis B virus (HBV) have also been reported in patients with ECR.^106,107^

#### Systemic disease

By far, there has been no evidence pointing to a direct link between systemic diseases and ECR. Thus, our understanding of the correlation between them is mainly based on case reports. A recent epidemiological study claimed that 2.4% of patients were diagnosed with systemic diseases.^61^ Bone metabolism disorder is by far the most commonly reported systemic disease related to ECR. Celiac disease, which is likely to cause deficiency of Vitamin D3, was discovered in a patient diagnosed with MCRR.^108^ Hypothyroidism was identified in a family, in which three members were diagnosed with ECR.^64^ In addition to systemic diseases, reports have also presented cases of ECR related to oral diseases, including peripheral odontogenic fibroma and oral focal mucinosis.^109,110^

Nevertheless, there has been diverse perspectives on the correlation between systemic disease and ECR. A case control study revealed that the control group had more systemic conditions than the ECR group. The only exception appeared in diabetes, which showed a significantly higher incidence rate among ECR patients.^66^ This could be attributed to the hypoxic cellular microenvironment induced by diabetes, which is favorable for osteoclastogenesis.^111^ Since our knowledge concerning the synergism between systemic disease and ECR mainly derives from sporadic cases, the underlying mechanism needs to be further elucidated.

#### Medication

Bisphosphonate (BSPs)-related ECR has been reported in a number of cases.^112^ Several studies on its mechanism have been carried out, most of which point to the proinflammatory property of BSPs. It is believed that amino-containing BSPs is capable of triggering the production of proinflammatory cytokines such as TNF-a, IL-1, and IL6, which could lead to activation of osteoclasts.^113–115^ Another factor contributing to resorption initiation is the prolonged skeletal half-life of BSPs, which has been estimated to be ~10 years.^116^ As thus, the importance of thorough dental assessments before, during, and after BSPs therapy is essential for the prevention of ECR.

Apart from BSPs, chemotherapy and tetracycline related ECR have also been shown in cases.^12,117^ However, according to a case control study, the difference between the control and ECR group regarding medication was not significant. In view of the limited number of cases related to medication, the correlation between medication and ECR calls for further research.^66^

## Conclusion and prospect

Despite being previously regarded as an uncommon disease, the number of case reports on ECR has been increasing rapidly in recent years. In the meantime, research progress on potential predisposing factors have provided significant reference for the prevention and diagnosis of ECR. Nevertheless, owing to a lack of foucs on its pathogenesis, our understanding of the etiology of ECR is deficient, which may explain why the majority of patients were diagnosed in advanced stages. In view of the difficulty in early diagnosis along with the undesirable outcome of ECR, a comprehensive understanding of its potential predisposing factors as well as its pathogenesis is essential. Summarizing the findings of recent cross-sectional studies and case reports may help overcome the bias brought about by different studies. Further research on etiology, especially the underlying mechanism of various predisposing factors, may provide pragmatic guidance for clinical practice.

## Supplementary information

Alterations in revised manuscript

manuscript with labels of revisions
