# Supplementary material for: External cervical resorption—a review of pathogenesis and potential predisposing factors
Source: Int J Oral Sci. 2021 Jun 10;13:19. doi: 10.1038/s41368-021-00121-9 (PMC8192751; doi:10.1038/s41368-021-00121-9)
Supplement: Supplementary file 1 — Alterations in revised manuscript [file 41368_2021_121_MOESM1_ESM.docx]

**Revisions in the manuscript**

We have meticulously modified the manuscript as requested by the reviewers’. All the suggestions were taken into account and we have revised the manuscript accordingly. We would like to express our genuine gratitude to the International Journal of Oral Science Editorial Office as well as the reviewers for offering constructive suggestions on our manuscript. The additional contents added to the revised manuscript are presented in highlighted form, and in the meantime the sections removed froom the former manuscript are presented in strikethroughs. The list of changes made in the revised manuscript is presented below.

1. Two sentences were revised in the abstract at page 2 line2 & 8-11.
2. Three senteces were revised in the “introduction” section at Page 2 line 24-27 & line 31-36, and page 3 line 12-15.
3. A sentence was revised at the second paragraph of the pathogenesis section at page 3, line 27-30.
4. The position of the “Histopathology” section and the “Mechanism” section were reversed according to the reviewer’s suggestion (page 3-4). The paragraph introducing the PRRS was removed at page 4.
5. The subtitle “Hypothesis of the initiation of ECR” was changed into “Cellular and molecular mechanisms of ECR” at page 5, line 4-5. Two additional paragraphs were added posterior to the subtitle at page 5.
6. Three sentences were revised at page 6, line 10-12 & line 30-32 & line 38.
7. The paragraph regarding macrophages was revised at page 7, line 1-4.
8. The first paragraph of the “potential predisposing factor” section was revised at page 8, line 2-8.
9. An additional sentence was added to the end of page 8 according to the reviewer’s suggestions.

(10) An additional paragraph was added at page 9 according to the reviewer’s suggestions.

(11) Two phrases were revised in page 10, line 1 & 12 according to the reviewer’s suggestions.

(12) An additional paragraph was added at page 10 according to the reviewer’s suggestions.

(13) The section regarding “Surgery” at page 13, line 31-39 was revised according to the reviewer’s suggestions.

(14) The section regarding “Undesirable oral hygiene” at page 14, line 12-20 was revised according to the reviewer’s suggestions.

(15) The sections regarding “Systematic disease” and “medication” at page 15 were revised according to the reviewer’s suggestions.

(16) Figure 1 in the previous manuscript was removed following the reviewers’ suggestions. On top of that, Fig2, Fig3, and Fig4 were modified so as to improve their quality of esthetics.

(17) An additional figure consisting of a schematic image as well as pathologcial sections was added to the manuscript at the *Pathology* section according to the reviewer’s request.
